# Modeling and optimization of radish root extract drying as *peroxidase* source using spouted bed dryer

**DOI:** 10.1038/s41598-021-93563-4

**Published:** 2021-07-13

**Authors:** Shahrbanoo Hamedi, M. Mehdi Afsahi, Ali Riahi-Madvar, Ali Mohebbi

**Affiliations:** 1 Department of Chemical Engineering, Shahid Bahonar University of Kerman, Kerman, Iran; 2 Department of Molecular and Cell Biology, Faculty of Basic Sciences, Kosar University of Bojnord, Bojnord, Iran

**Keywords:** Chemical engineering, Chemistry

## Abstract

The main advantages of the dried enzymes are the lower cost of storage and longer time of preservation for industrial applications. In this study, the spouted bed dryer was utilized for drying the garden radish (*Raphanus sativus* L.) root extract as a cost-effective source of the *peroxidase* enzyme. The response surface methodology (RSM) was used to evaluate the individual and interactive effects of main parameters (the inlet air temperature (T) and the ratio of air flow rate to the minimum spouting air flow rate (Q)) on the residual enzyme activity (REA). The maximum REA of 38.7% was obtained at T = 50 °C and Q = 1.4. To investigate the drying effect on the catalytic activity, the optimum reaction conditions (pH and temperature), as well as kinetic parameters, were investigated for the fresh and dried enzyme extracts (FEE and DEE). The obtained results showed that the optimum pH of DEE was decreased by 12.3% compared to FEE, while the optimum temperature of DEE compared to FEE increased by a factor of 85.7%. Moreover, kinetic parameters, thermal-stability, and shelf life of the enzyme were considerably improved after drying by the spouted bed. Overall, the results confirmed that a spouted bed reactor can be used as a promising method for drying heat-sensitive materials such as *peroxidase* enzyme.

## Introduction

Enzymes are protein catalysts extensively used in various industries. However, their sensitivity to heat, especially in the form of liquid, is a major application problem^1^. In this regard, various methods including the use of osmolytes^2^, mutagenesis^3^, immobilization^4–7^, and drying^8^ have been developed to increase the thermal as well as the storage stability of the enzymes. One of the impressive and applicable methods for improving enzyme stability is enzyme drying. This technique significantly reduces the initial solution weight as well as packaging and transportation costs^9^.

One of the oxidoreductase heat-sensitive enzymes is *peroxidase* that catalyzes a wide variety of reactions in the presence of peroxides. According to the literature, *peroxidase* has various applications in industries, such as the removal of the phenolic pollution from the wastewaters, decolorization of the synthetic paints, bio pulping and biobleaching, analysis and diagnostic kits, design and construction of biosensors, and synthesis of aromatic amines, phenolic compounds and polymers^10^. *Peroxidase* can be extracted from plants and animals or produced by microorganisms in a fermenter^10,11^. The enzyme extraction from the plant sources has considerable advantages including low production cost and renewability^12^. *Peroxidase* has been extracted from plants such as *Brassica rapa*, *Lycopersicon esculentum*, *Raphanus sativus* L., and *Brassica oleracea*^13–16^.

Few papers have been published about enzyme drying by spouted bed^17^. Many studies have been published on spouted bed drying in other fields of studies such as food industries and pharmaceticals^18,19^.

According to relevant literature reviews, several methods have been applied for the preparation of enzyme powder, including freeze drying^20^, spray drying^21–23^, and spouted bed drying^17^. Spray drying of alpha-amylase^20^, protease^22^, lipase^24^, freeze-drying of alpha-amylase^20^ and spouted bed of lipase^17^ are examples of enzyme drying. Among the mentioned dryers, the spouted bed can be used as an effective drying equipment, due to its advantages such as low cost^25^, ability to work at low temperature, and high volumetric evaporation rates under identical thermal conditions^26^.

In this study, *Peroxidase* was extracted from garden radish (*Raphanus sativus* L.) as a low-cost source^27^ and a full factorial experimental design was employed to evaluate the influence of input operating variables (inlet air temperature and flow rate) on drying by spouted bed. After process optimization, an enzymatic reaction in the presence of the fresh enzyme extract (FEE) and dried enzyme extract (DEE) was carried out to evaluate the enzyme catalytic activity. Kinetic parameters of the enzymatic reaction (*V*_*max*_ and *K*_m_) were obtained using Michaelis–Menton (M–M) equation. Moreover, the thermal stability and shelf-life of FEE and DEE were determined and compared. To the best of our knowledge, no experimental or modeling study has been investigated on the *peroxidase* drying by a spouted bed.

## Materials and methods

### Materials

The roots of the garden radish were purchased from a local market, Kerman, Iran. Also, hydrogen peroxide (H_2_O_2_), 3,3′,5,5′-tetramethylbenzidine (TMB), dipotassium phosphate (K_2_HPO_4_), Monopotassium phosphate (KH_2_PO_4_), bovine serum albumin (BSA), and Coomassie Brilliant Blue G-250 (CBBG) were all purchased from Sigma-Aldrich (St. Louis, MO, USA). Double distilled water was used in all experiments.

### Enzyme extraction and quantification

The enzyme extract was obtained using the technique defined by Riazi et al.^15^ with a slight modification. The garden radish roots were peeled and the extract was prepared with a juicer. The slurry solution was then filtered, homogenized, and stored in a freezer (− 20 °C) until used. The total protein concentration of the extract was estimated according to Bradford’s method^28^ using bovine serum albumin (BSA) as standard. All experiments complied with relevant institutional, national, and international guidelines and legislation.

### Drying procedure

**Figure 1:**
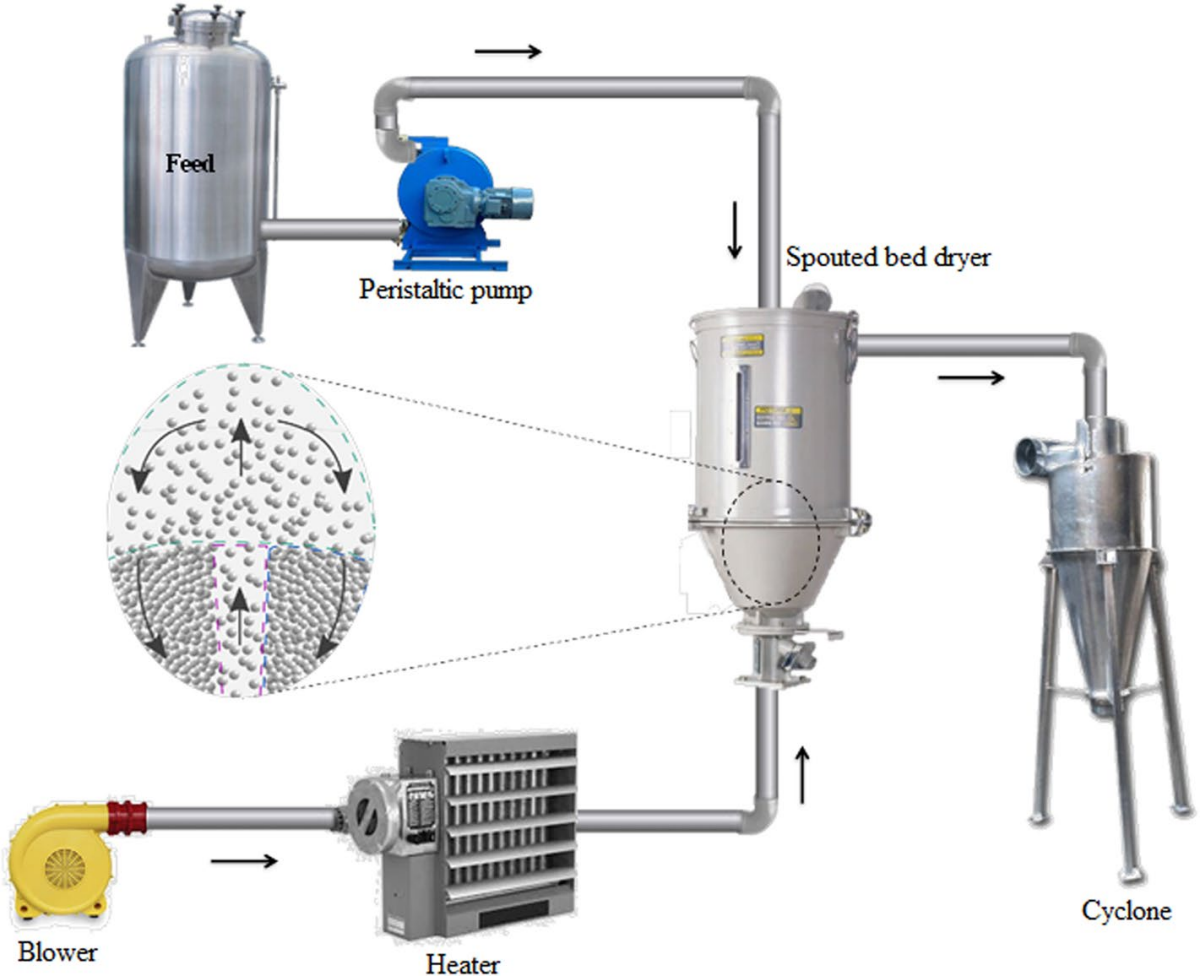
Schematic diagram of the spouted bed dryer.

The drying procedure was performed in a spouted bed consisting of a plexiglass cylindrical column with an inner diameter of 90 mm and a height of 300 mm that was connected to the conical base of the dryer (the internal angle of 60° and the inlet orifice diameter of 15 mm). Details of the used spouted bed and experimental procedure can be found in our previous published paper^18^. The main components of the system were a heater with two elements by total power of 8 kW, a blower with a power of 2.2 kW (GREENCO 2RB, China) which was equipped with a three-phase inverter, a peristaltic pump (WPX-1, Welco Co., Japan), an air flow meter, and a cyclone (Fig. 1). The temperature and humidity of the inlet and outlet air were controlled using the sensors installed at different points. Glass granules (diameter 3 mm, sphericity 1, density 2343.1 ± 9.8 kg/m^3^) were considered as the inert materials to be a carrier for the liquid film, a conductive heat transfer medium, and a mechanical cleaner of the bed^29^. When the outlet air temperature showed a constant value by Data Logger (Testo, T4 176), a constant feed flow rate with 1.22 ± 0.09 ml/min was dropped on the glass beads (360 g) using the peristaltic pump. The drying process was performed according to convective and conductive heat transfer in the bed. At the end of each experiment, the produced powder at the bottom of the cyclone was collected to analyze the enzyme activity. After each test, the granules were removed from the bed, washed several times, and dried for further use.

### Minimum spouting air flow rate

**Figure 2:**
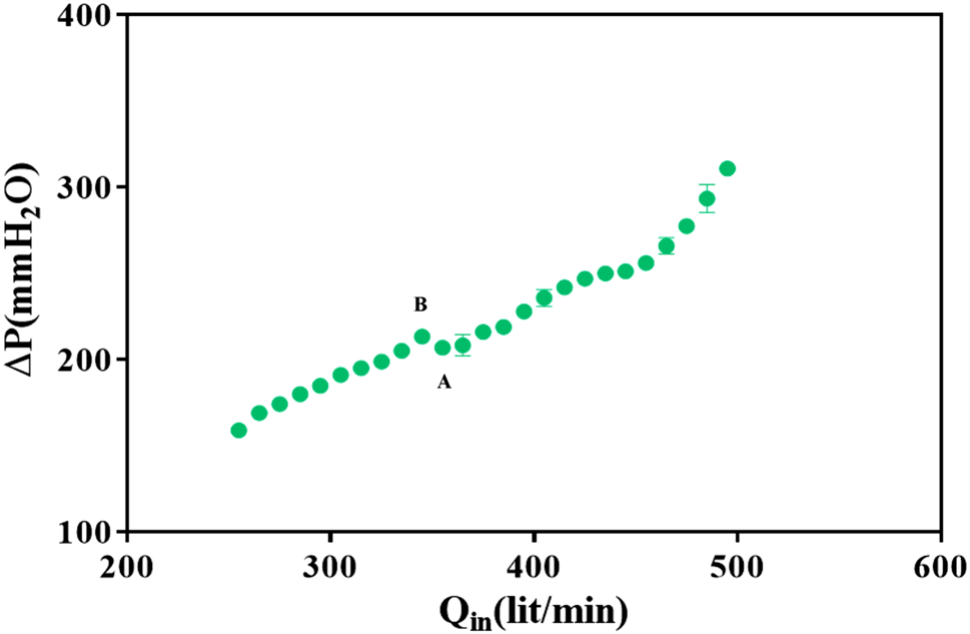
Pressure drop (∆P) versus the inlet air flow rate (Q_in_).

The minimum air flow rate to spout the inert glass beads (Q_min_) was determined according to the method developed by Mathur and Epstein^30^ from the curve of the bed pressure drop versus the air flow rate (Q_in_). It was obtained experimentally by slowly decreasing the air flow rate. Point A in Fig. 2 represents the minimum spouting condition where the bed remains in the spouted state. A slight reduction of air velocity at this condition causes the spout to collapse and the pressure drop to rise suddenly to point B. The bed pressure drop and air flow rate were measured using a digital differential manometer (Testo 510i) and a rotameter, respectively. Each experiment was repeated three times to provide an acceptable level of reliability.

### Residual enzyme activity (REA)

*Peroxidase* activity was determined according to the procedure described by Krainer et al.^31^. The reaction was started by the addition of 1 mM H_2_O_2_ to a solution including 0.6 mM TMB, 50 mM potassium phosphate buffer, and the enzyme extract. The optical density was measured at 653 nm (extinction coefficient of oxidized TMB was 3.9 × 104 mol/cm) by a spectrophotometer (Cary 50, Australia) for 180 s. The specific activity is defined as the ratio of the enzyme activity to the total protein amount^32^. REA was calculated as the ratio of the specific activity of the enzyme powder to the specific activity of the enzyme extract, as shown in Eq. (1) ^17^:

1 $${\text{REA = }}\frac{{{\text{Specific activity of the powder enzyme}}}}{{{\text{Specific activity of the extract enzyme }}}} \times 100.$$

### Experimental design

To evaluate the effect of drying conditions on the REA, the experimental design was conducted at different inlet air temperatures and flow rates. Minitab 17 software was used to design the experiment and analyze the obtained data. A full factorial design, considering the inlet air temperature at three levels of 50, 60, 70 °C and the dimensionless air flow rate at two levels of 1.2 and 1.4 resulted in 18 experiments that were performed with three times replication. To predict REA at different conditions, a first-order polynomial relationship relative to relevant variables has been presented in Eq. (2):

2 $$REA\% = \beta _{0} + \beta _{1} {\rm X}_{1} + \beta _{2} {\rm X}_{2} + \beta _{{12}} {\rm X}_{1} {\rm X}_{2} ,$$

where, REA% is the response of model, β_0_, β_i_, and β_ij_ are the constant, linear, and interaction coefficients of the models, respectively. X_1_ and X_2_ are the inlet air temperature and dimensionless air flow rate (the ratio of Q_in_ to Q_min_), respectively^33^. To achieve the enzyme maximum residual activity, the obtained data were analyzed using RSM. Enzyme powder, obtained under the best drying conditions, was utilized in the subsequent experiments.

### The catalytic activity of the enzyme

To evaluate the activity of the dried *peroxidase* enzyme, the reaction of 0.6 mM TMB with 1 mM hydrogen peroxide in the presence of the DEE and FEE was carried out^31^. The specific activity of the enzyme can be changed by the temperature, pH, and concentration of the substrate. In order to find the optimum value of pH, *peroxidase* activity was measured at various pH values from 3 to 10 at ambient temperature. The optimum temperature of the reaction was also determined by the measurement of the FEE and DEE activity during the reaction at different temperature ranges (10–85 °C) under the optimum pH^34^.

### Kinetic parameters of the enzymatic reaction

Under the obtained optimum conditions, the reaction between TMB and hydrogen peroxide was carried out in the presence of the *peroxidase* and different concentrations of the substrate (0.06–0.6 mM TMB) and 1 mM H_2_O_2_^34^. The rate of the reaction at different conditions can be calculated by Michaels–Menten (M–M) equation. By inverting this equation, a linear relationship can be obtained which is called Line weaver-Burke Equation (Eq. (3)). The kinetic parameters of the reaction can be determined from the slope and intercept of this line^35^.

3 $$\frac{1}{{V_{s} }} = \frac{1}{{V_{{\max }} }} + \frac{{K_{m} }}{{V_{{\max }} S}}.$$

In this equation, *V*_s_ is the reaction rate (µM/min mg), *V*_max_ is the maximum reaction rate (µM/min.mg), and *K*_m_ is the M–M equation constant (mM). The product of the mentioned reaction was a colored compound and the color changed with the reaction progress. Therefore, the intensity of light absorption could be determined at different times by a spectrophotometer. Finally, the concentration of the product (C_P_) at different times was determined from the Beer–Lambert equation. In the plot of C_P_ versus time, several slopes can be obtained at different initial substrate concentrations. The reaction rate (*V*_*s*_, µM/min.mg) was calculated by dividing the slope of plots by the amount of the protein content.

### Peroxidase thermal-stability and self-life during storage

To determine the possible variation in enzyme thermal-stability, the DEE and FEE were incubated for 10 min at different temperature ranges (25–80 °C), and then after 5 min incubation at room temperature, enzyme activity was measured^2^. The shelf-life of DEE and FEE during storage was followed by the determination of the changes in the enzyme activity during storage at − 4 °C.

## Results and discussion

### Minimum spouting air flow rate

According to Fig. 2, the minimum spouting air flow rate was observed to be 355 l/min. The ratio of Q_in_ to Q_min_ in each experiment is a dimensionless parameter for investigating the effect of drying on the residual activity**.**

### Experimental design

#### Full factorial experiment design methodology and model consequence for REA

The full factorial design matrix with two independent variables and results obtained from empirical experiments and model prediction are shown in Table 1. Based on the experimental data, the following equation Eq. (4) can be expressed as a model that shows a correlation between the REA and the operating variables:

4 $${\text{REA}}\% {\text{ }} = {\text{ 2}}0{\text{7}}.{\text{4 }} - ~{\text{3}}.{\text{493 X}}_{{\text{1}}} - ~{\text{95}}.{\text{1 X}}_{{\text{2}}} + ~{\text{1}}.{\text{992 X}}_{{\text{1}}} {\text{X}}_{{\text{2}}} .$$

**Table 1:** The results of full factorial design relevant to the residual enzyme activity.

Run	T (°C)	Q	The residual enzyme activity (%)		Error (%)
			Experimental	Predicted	
1	50	1.4	42.0	39.1	7.4
2	60	1.4	32.9	32.0	2.8
3	70	1.2	17.9	16.1	11.2
4	70	1.4	27.5	25.0	10.0
5	60	1.4	33.5	32.0	4.7
6	50	1.2	35.6	38.2	6.8
7	60	1.2	30.2	27.1	11.4
8	70	1.4	23.7	25.0	5.2
9	60	1.2	27.3	27.1	0.7
10	50	1.2	41.8	38.2	9.4
11	70	1.4	22.5	25.0	10.0
12	70	1.2	14.8	16.1	8.1
13	70	1.2	14.4	16.1	10.6
14	60	1.4	33.4	32.0	4.4
15	50	1.2	35.6	38.2	6.8
16	50	1.4	35.9	39.1	8.2
17	50	1.4	35.3	39.1	9.7
18	60	1.2	25.9	27.1	4.4

To evaluate the importance and suitability of the obtained model, the analysis of variance (ANOVA) was carried out and the results are shown in Table 2. From the table, it can be concluded that the regression model had a high determination coefficient (R^2^ = 0.95).

**Table 2:** Analysis of variance relevant to the residual enzyme activity.

Source of variations	DF	Adjusted Mean Square	F-value	p-value
Regression model	3	378.726	86.79	0.000
Linear	2	544.288	124.73	0.000
T	1	981.021	224.81	0.000
Q	1	107.556	24.65	0.000
T*Q	1	47.601	10.91	0.005
Error	14	4.364	–	–
Lack-of-Fit	2	1.844	0.39	0.688
Pure Error	12	4.784	–	–
R^2^ = 94.90%, R^2^adj = 93.80%, R^2^pred = 90.59%				

As can be observed, all the terms of the model (the linear and interaction terms) have a p-value less than 0.05, which means that both terms are significantly effective on REA^36,37^. Moreover, the calculated F-value for the regression model is meaningfully higher than the acquired F-distribution, which demonstrates the predicted strength of the fitted model^38^. According to this result, the appropriateness and credibility of the model are confirmed for the simulation of the REA.

**Figure 3:**
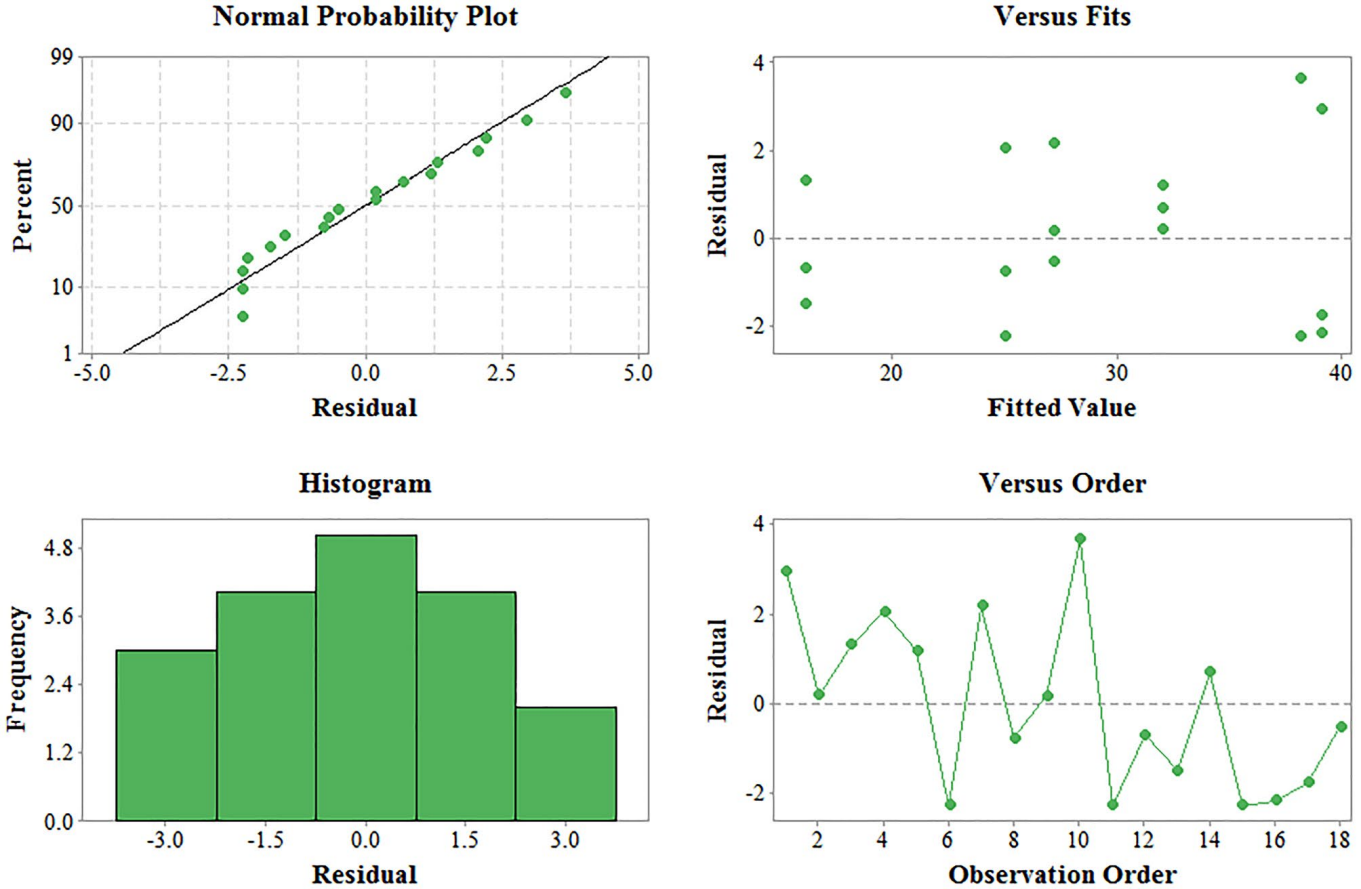
Residual plots for residual *peroxidase* activity.

The residual analysis was used to further check the model adequacy. This analysis indicates the difference between the experimental data and the computed data by the model. Figure 3 illustrates the residual plots related to the residual data which was generated by 18 experimental runs. As can be seen, the tendency in all residual plots follows a normal distribution. Accordingly, it can be stated that the obtained model has good adequacy and satisfactoriness.

Regression analysis of the experimental data including T-values, regression coefficients, and p-values are shown in Table 3. The p-values were used as a tool to evaluate the significance and importance of each coefficient. The higher amount of the T-value and lower amount of the p-value reveal that the corresponding coefficient is more significant and has a greater effect on the response. The results demonstrated that the inlet air temperature (p-value = 0.0), flow rate (p-value = 0.0), and their interaction (p-value = 0.005) were significant parameters in *peroxidase* drying process. As seen, some of the coefficients are positive and some are negative. The positive or negative coefficients display that these parameters will increase or decrease the response, respectively. Among the discussed parameters, the T had a negative effect on REA while Q had a positive effect.

**Table 3:** Estimated regression coefficients, T- and p-values.

Terms	Coded coefficient	T-value	p-value
β_0_	29.578	60.07	0.000
β_1_	− 9.042	− 14.99	0.000
β_2_	2.444	4.96	0.000
β_1_ × β_2_	1.992	3.30	0.005

#### Effect of the inlet air temperature and flow rate on the REA

**Figure 4:**
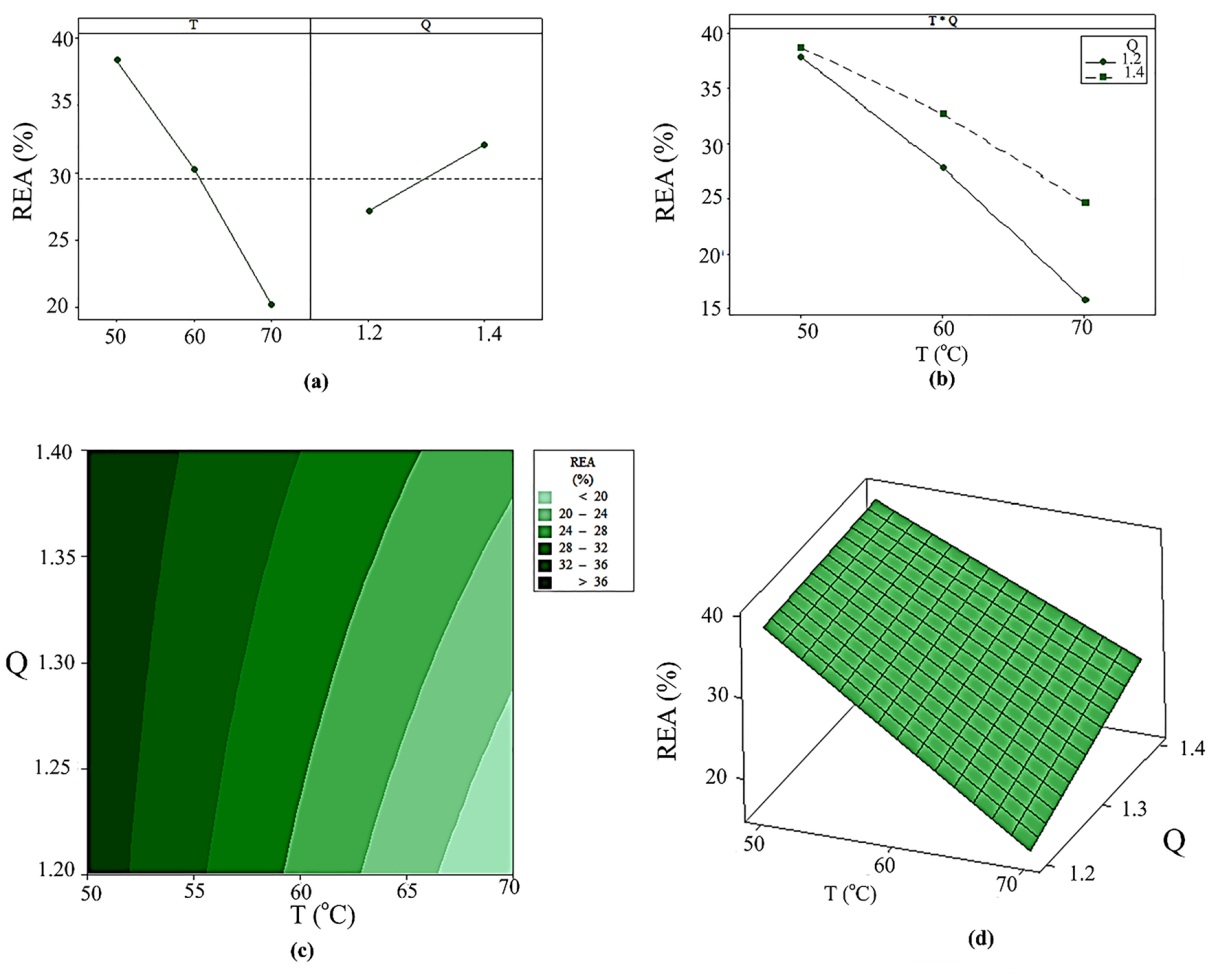
(**a**) The effect of the inlet air temperature (T) and the ratio of air flow rate to the minimum spouting air flow rate (Q) on the residual enzyme activity (REA), (**b**) the interaction effect of T and Q on the REA, (**c**) the contour and (**d**) surface plot of the REA (%).

The effect of the inlet air temperature and the flow rate on the REA are illustrated in Fig. 4. As it is obvious from Fig. 4a, the flow rate has a positive effect and REA increases with the increment of the flow rate at the constant values of T. Conversely, the inlet air temperature has a negative effect on REA and an increase in the inlet air temperature from 50 to 70 °C leads to a decrease in the REA. The results in Fig. 4a demonstrate that at higher inlet air temperatures, the positive effect of the inlet air flow rate was more noticeable on REA, compared to lower temperatures. Furthermore, the slope of lines in Fig. 4b is different, which indicates the importance of the effect of each factor on the REA.

To further investigate the influence of each factor on REA and to discover the relative optimum range, the contour and surface plots were obtained. Figure 4c,d depict the contour and surface plots as a function of two input operating factors. The darker area in Fig. 4c indicates the higher amount of REA. Figure 4d represents the effect of the temperature and the air flow rate on REA; the maximum of these parameters occurred at T = 50 °C and Q = 1.4.

According to the obtained results, it was found that by increasing the temperature, the REA decreased, which agrees with earlier research^22,23^. Indeed, increasing the temperature can change the chemical structure of the enzyme and therefore reduce the enzyme activity. On the other hand, at the higher inlet air flow rate, due to the lower residence time of the powder, the enzyme was less affected by the heat and then the amount of the REA increased. Under the best drying conditions (T = 50 °C and Q = 1.4), the REA was obtained to about 38.7%.

### Effect of drying on the catalytic activity of the enzyme

**Figure 5:**
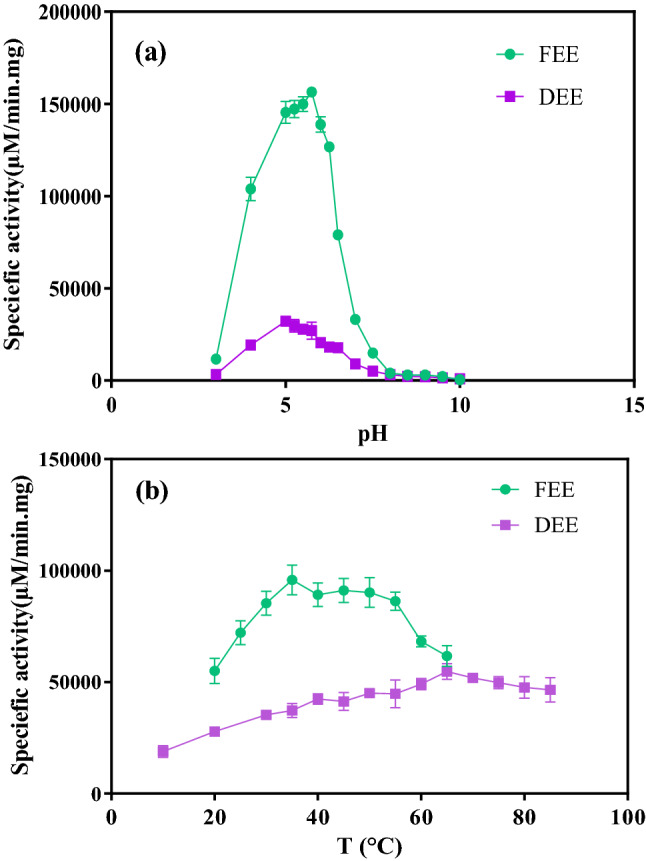
The optimum conditions of the enzymatic reaction in the presence of the freshly extracted enzyme (FEE) and the dried extracted enzyme (DEE) (**a**) experiment at the ambient temperature and different pH ranges (3–10) (**b**) at the optimum pH and different temperature ranges (10–85 °C).

The effect of pH on the enzymatic reaction at 25 °C has been shown in Fig. 5a. By increasing the pH value from 3 to about 5.75 for FEE and from 3 to 5 for DEE, the specific activity of the enzyme was enhanced. Further increase in pH value reduced the residual enzyme activity. As the result, it was inferred that the optimum values of 5.75 and 5 can be considered for FEE and DEE, respectively. The effect of temperature on the enzymatic reaction at the obtained optimum pH has been shown in Fig. 5b. The optimum temperature of the reaction catalyzed by FEE and DEE was observed at about 35 °C and 65 °C, respectively. As can be seen in this figure, although the reduction in activity owing to drying is noticeable, the optimum temperature of the enzymatic reaction has been increased in the presence of *peroxidase* as powder form.

As shown in Fig. 5, the activity of the enzyme has been affected upon drying. The optimum pH shifted to the acidic pH in DEE compared with the FEE. Interestingly, the optimum temperature of the reaction for DEE is increased by more than 1.8-folds.

### Effect of drying on the kinetics of the peroxidase reaction

To evaluate the influence of initial concentrations of TMB on the product concentration of FEE and DEE, the experiments were carried out in the presence of different concentrations of TMB (0.06–0.6 mM) and the obtained results are presented in Fig. S1 (Supporting file). As evident, at initial times of reaction (about until 3 min), the slope of each line is constant and independent of the substrate concentration. Therefore, for each line, an initial rate was obtained in accordance with the initial substrate concentration. The reaction rates were obtained by dividing the obtained slope by the amount of protein content. The obtained results from Fig. S1, the initial slopes, and the reaction rates are presented in Table 4 for the dried enzyme and the fresh extracted one.

**Table 4:** The slopes and the reaction rates at different initial concentrations of the substrate.

C_0,TMB_ (mM)	Slope_DEE_ (µM/min)	Slope_FEE_ (µM/min)	Reaction rate (Vs_DEE_) (µM/min mg)	Reaction rate (Vs_FEE_) (µM/min mg)
0.06	1.44 ± 0.07	0.30 ± 0.00	9234 ± 419	22,087 ± 109
0.09	2.11 ± 0.19	0.42 ± 0.02	13,508 ± 1197	30,439 ± 1135
0.12	2.62 ± 0.21	0.46 ± 0.03	16,754 ± 1338	33,626 ± 2051
0.15	3.24 ± 0.03	0.55 ± 0.051	20,723 ± 172	40,000 ± 3736
0.18	3.69 ± 0.20	0.68 ± 0.01	23,613 ± 1290	49,487 ± 842
0.2	4.36 ± 0.29	0.69 ± 0.02	27,938 ± 1837	50,329 ± 1684
0.4	5.94 ± 0.19	0.93 ± 0.00	38,018 ± 1245	68,095 ± 256
0.6	6.78 ± 0.23	0.97 ± 0.04	43,418 ± 1446	70,842 ± 2637

**Figure 6:**
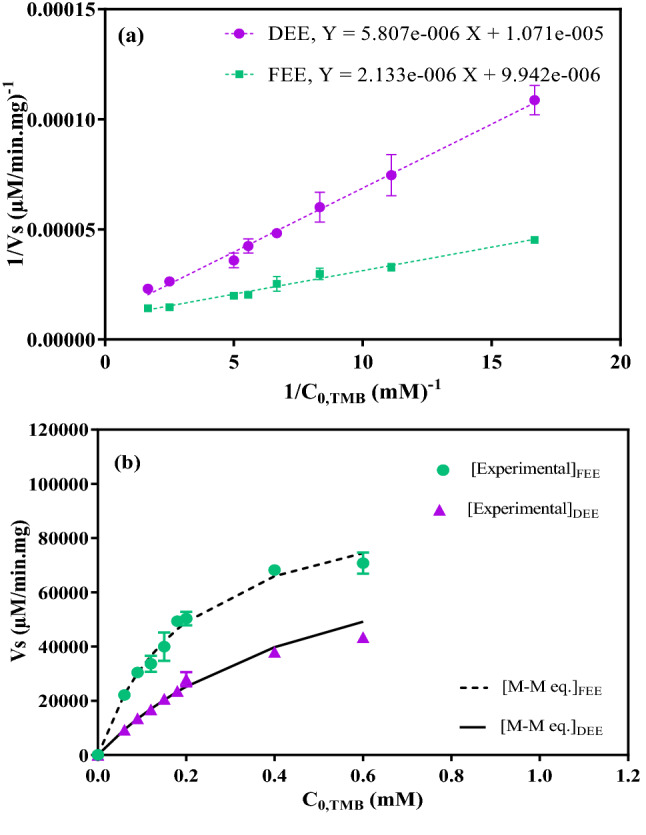
(**a**) Lineweaver–Burke Equation parameters “Eq. (4)” (**b**) The initial reaction rate obtained from the M–M equation and the experimental data at different substrate concentrations using the dried extracted enzyme (DEE) and freshly extracted enzyme (FEE).

According to Eq. (4), by drawing the inverse of the reaction rates versus the initial substrate concentrations, the M–M parameters (*K*_m_ and *V*_max_ constants) were obtained (Fig. 6a) as presented in Table 5. After obtaining the constants, the rate of the reaction could be calculated from the M-M equation for different concentrations. The calculated rates from the M–M equation and the experimental data are presented in Fig. 6b. As can be seen, a clear decrease in enzyme activity was observed in DEE. The calculations showed that the FEE activity was reduced by 48.9%, on average, after the drying process.

**Table 5:** The obtained kinetic parameters of the dried extracted enzyme (DEE) and freshly extracted enzyme (FEE).

K_m, DEE_ (mM)	K_m, FEE_ (mM)	*V*_*max*, DEE_ (µM/min mg)	*V*_*max*, FEE_ (µM/min mg)
0.54	0.21	93,370.68	100,583.38

In addition to the physicochemical properties, the kinetic parameters of *peroxidase* were also affected upon enzyme drying, which may be due to *peroxidase* structural changes. Based on the results, because of drying, the affinity of the enzyme to substrate increased and the maximum reaction rate (*V*_max, DEE_) decreased. Indeed, amino hydrophilic acids are in the exterior structure of the enzyme, creating hydrogen bonds between the enzyme and the water molecules^39^. As a result of drying, the aqueous medium around the enzyme is removed, causing hydrogen bonds to break down^40^, which leads to an increase in the salt concentration followed by a change in the electrostatic interaction between charged amino acids^41^. These changes may cause a variation in the enzyme. Changes in kinetic properties as a result of enzyme structure variation during drying have been reported previously^2–6^.

### Effect of drying on the thermal-stability and shelf-life during storage

**Figure 7:**
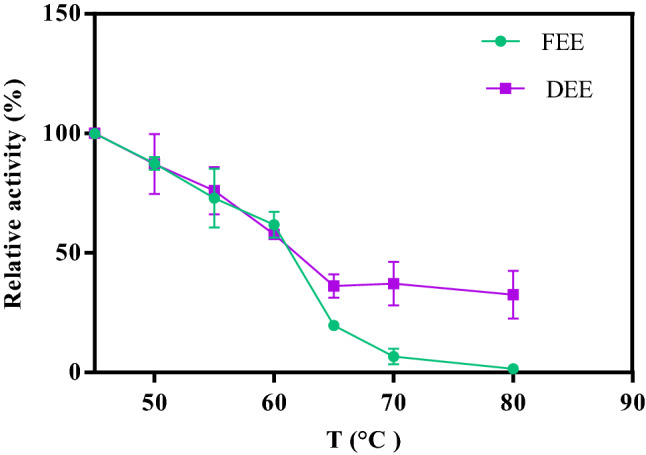
Thermal stability of the *peroxidase* as the dried extracted enzyme (DEE) and freshly extracted enzyme (FEE).

Relative activity at each temperature indicates the ratio of the specific *peroxidase* activity at a specific temperature to the maximum specific *peroxidase* activity which is the specific *peroxidase* activity at 45 °C^42^. As can be seen in Fig. 7, the enzyme powder showed more stability toward heating compared to the extract one. For instance, at 80 °C, enzyme extract activity tended to be zero, while more than 30% of the activity of enzyme powder was remained. Moreover, the shelf-life of the powder and extract was examined during storage. The *peroxidase* powder maintained 87.24% of its initial activity after storage at − 4 °C for 9 months while the *peroxidase* extract lost about 20% of its initial activity after one month of storage at the same temperature.

In accordance with Fig. 7, the thermal stability of the enzyme powder was improved during drying in the spouted bed. Although the increase of enzyme thermal-stability was reported by several methods^2,41^, to the best of our knowledge, this is the first time that the enhancement of *peroxidase* thermal-stability was reported by a drying process. The shelf-life of the DEE during storage was also improved owing to drying in the spouted bed, which is in agreement with the results of the enzyme drying in spray dryer^43^. Generally, improvement of optimum temperature, thermal stability, and shelf-life during storage has several advantages for the utilization of dried enzymes in industries which results in low-cost storage and long-time preservation as well as its use at higher temperatures^1,9^.

As shown in the results, the enzyme powder is active and more stable in the presence of heat, but the residual activity was relatively low. Therefore, further studies are needed to improve the residue activity as well as the other physicochemical properties of the dried enzyme by the use of protective or stable agents. Until now, several studies have been conducted and used some protective agents that are presented in Table 6.

**Table 6:** Effect of stabilizer agents on the residual enzyme activity in different studies.

Enzyme type	Dryer type	Operational factors	Residual enzyme activity (%)	Stabilizer or adjuvant	Ref.
Lipase	Spray dryer	Inlet temperature of drying gas (86.4 to 153.6 °C), concentration of the drying adjuvant (1.95 to 12.05%), mass flow rate of the enzymatic extract (2.63 to 9.36 g/min)	23–100	Maltodextrin β-cyclodextrin	Costa-Silva et al.^44^
α-amylase	Spray dryer	inlet drying air temperature (160–220 °C), feed ratio speed (1.7–0.4 cm^3^/s)	51.9–91.8	Maltodextrin	Samborska et al.^23^
Lipase	Spray dryer	Type of adjuvants (63–100%)-without adjuvants	6.73–100 Without adjuvants = 0	Lactose β-cyclodextrin, maltodextrin, mannitol, gum arabic, trehalose	Costa-Silva et al.^24^
Lipase	Spray dryer	Type of adjuvants, Inlet temperature of drying gas (100,153.6 °C), feed flow rate (4–9.36 g/min)	87.7–99.6	β-cyclodextrin, Lactose, Maltodextrin	Costa-Silva et al.^45^
Protease	Spray dryer	Type of adjuvants, Inlet temperature of drying gas (70–130 °C)	79–94	Glucose, maltodextrin	Namaldi et al.^22^
Lipase	Spouted bed	Type of agricultural by-products	83.6–179.1	Rice husk, corn stover, sugarcane, bagasse green, coconut fiber, corncob	Costa-Silva et al.^17^

## Conclusion

The enzymes can be extracted directly from the natural resources of plants and animals; however, due to the short shelf-life of fresh enzymes, these materials should be dried. In this research, the root ingredients of garden radish which is a rich source of *peroxidase* were extracted and the resulting paste was dried using the spouted bed dryer with inert glass beads. The effect of important operating factors (the inlet air temperature and the air flow rate) and their interaction on the residual activity of enzyme were established by full factorial experimental design methodology for the first time. The ANOVA analysis indicated that the inlet air temperature, contrary to the air flow rate, had a negative effect on the residual activity of the enzyme. The obtained results indicated a reduction of the enzyme activity after the drying process which can be due to a change in *peroxidase* structure. After evaluating the optimum values of operating factors (T = 50 °C and Q = 1.4), the effect of the drying process on the thermal-stability and kinetics of the *peroxidase* was investigated. The kinetic studies were carried out according to the Michaelis–Menton (M–M) equation, and *V*_*max*_ and *K*_m_ of enzymatic reaction were obtained for different TMB concentrations. According to the stability studies, it was confirmed that spouted bed drying of *peroxidase* had an impressive effect on the thermal-stability and shelf-life which improved up to 50% compare to the fresh enzyme. Overall, it could be concluded that the spouted bed dryer can be a promising method -as a successful unit operation for drying the heat-sensitive materials such as enzymes.

## Supplementary Information

Supplementary Information.
