# Supplementary material for: Modeling and optimization of radish root extract drying as peroxidase source using spouted bed dryer
Source: Sci Rep. 2021 Jul 13;11:14362. doi: 10.1038/s41598-021-93563-4 (PMC8277770; doi:10.1038/s41598-021-93563-4)
Supplement: Supplementary file 1 — Supplementary Information. [file 41598_2021_93563_MOESM1_ESM.docx]

**Modeling and optimization of radish root extract drying as *peroxidase* source using spouted bed dryer**

Shahrbanoo Hamedi^a^, M. Mehdi Afsahi^a*^, Ali Riahi-Madvar^b*^, Ali Mohebbi^a^

^a^ Department of Chemical Engineering, Shahid Bahonar University of Kerman, Kerman, Iran

^b^ Department Cell and Molecular Biology, Kosar University of Bojnord, Bojnord, North Khorasan Province, Iran

^*^Corresponding Email Addresses: [afsahi@uk.ac.ir](mailto:afsahi@uk.ac.ir), riahi.ali@gmail.com


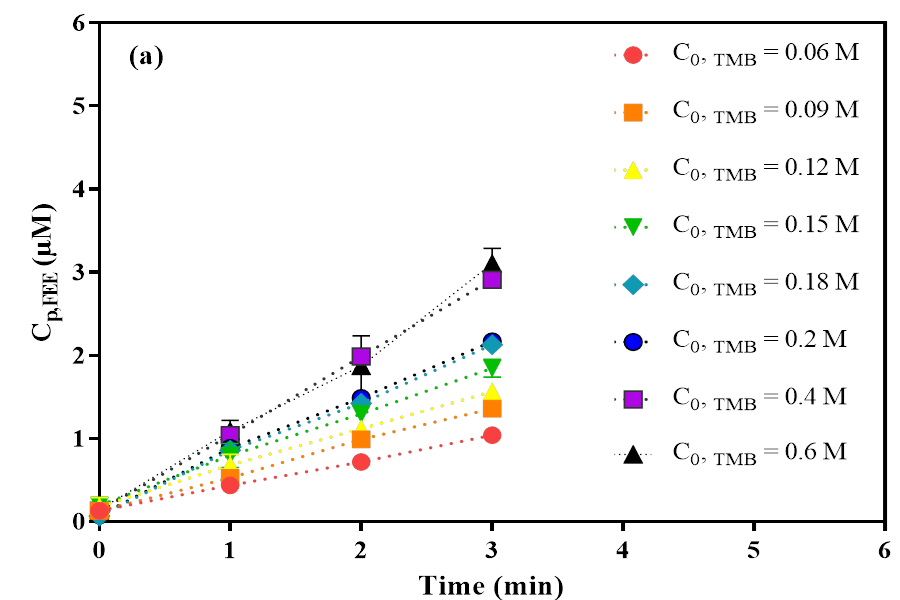


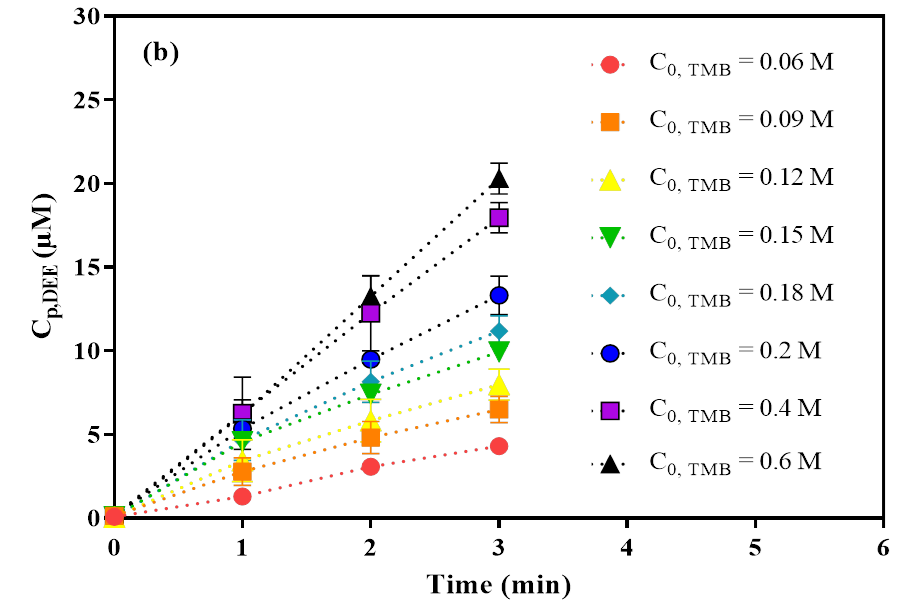


**Fig. 1S.** The concentration of the colored product versus time at different initial concentrations of the substrate (a) for the freshly extracted enzyme (FEE), and (b) for the dried extracted enzyme
